# Isolated Fallopian Tube Torsion in Adolescents

**DOI:** 10.1155/2013/341507

**Published:** 2013-10-22

**Authors:** S. Rajaram, S. Bhaskaran, S. Mehta

**Affiliations:** Department of Obstetrics and Gynaecology, Guru Teg Bahadur Hospital and University College of Medical Sciences, Delhi 110095, India

## Abstract

*Background*. Fallopian tube torsion is a rare cause of acute abdomen, occurring commonly in females of reproductive age. It lacks pathognomonic symptoms, signs, or imaging features, thus causing delay in surgical intervention. *Case*. We report two cases of isolated fallopian tube torsion in adolescent girls. In the first case a 19-year-old patient presented with acute pain in the left iliac region associated with episodes of vomiting for one day and mild tenderness on examination. Laparoscopy revealed left sided twisted fallopian tube associated with hemorrhagic cyst of ovary. The tube was untwisted and salvaged. In another case an 18-year-old virgin girl presented with similar complaints since one week, associated with mild tenderness in the lower abdomen and tender cystic mass on per rectal examination. On laparoscopy right twisted fallopian tube associated with a paratubal cyst was found. Salpingectomy was done as the tube was gangrenous. *Conclusion*. Fallopian tube torsion, though rare, should be considered in women of reproductive age with unilateral pelvic pain. Early diagnostic laparoscopy is important for an accurate diagnosis and could salvage the tube.

## 1. Introduction

Isolated torsion of fallopian tube is a rare entity occurring in 1 in 1.5 million women [1]. It is a surgical emergency, where prompt diagnosis and timely surgical intervention are vital to salvage the fallopian tube. It is rarely diagnosed preoperatively due to its rarity, lack of definitive diagnostic signs, and similarity to other cases of acute abdomen. We are reporting two cases in adolescent girls, where early intervention could salvage the tube in one of them.

## 2. Case

**Figure 1:**
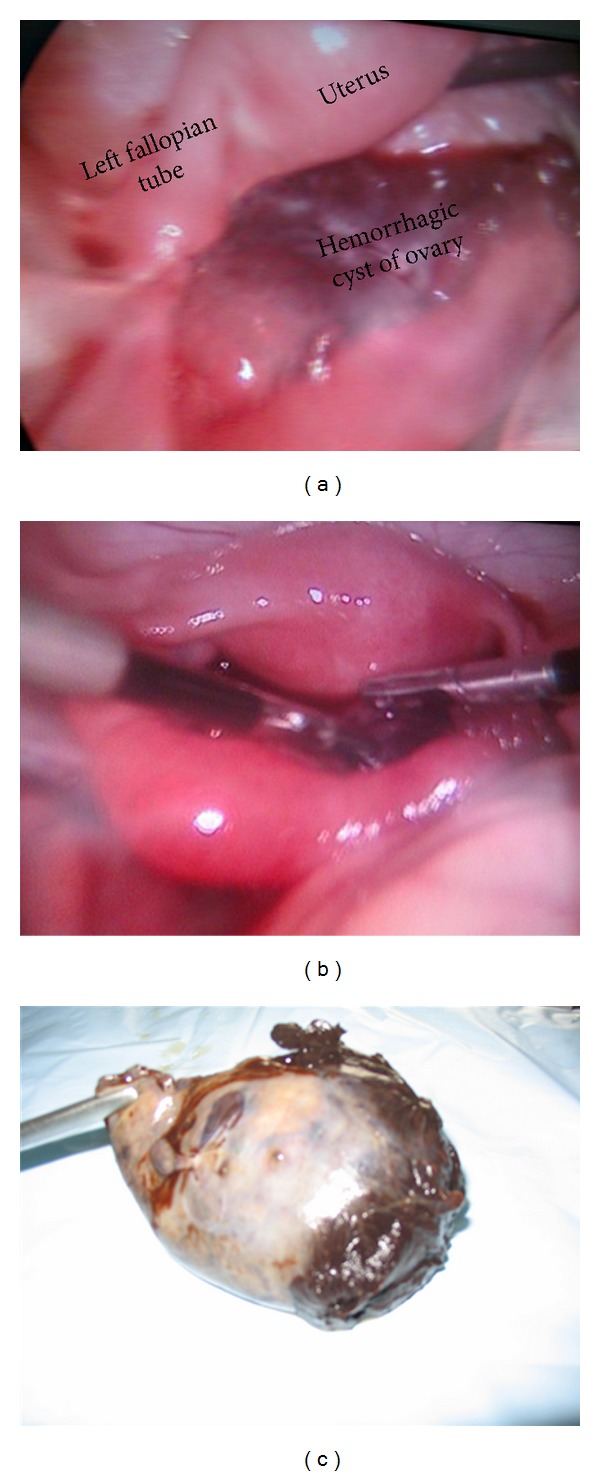
(a) Twisted left fallopian tube (arrow) with hemorrhagic cyst of ovary. (b) Left fallopian tube after untwisting. (c) Right fallopian tube, gangrenous.

In the first case a 19-year-old girl presented with complaints of pain in the left lower abdomen since one day associated with episodes of vomiting. Her menstrual cycles were regular. On abdominal and per rectal examination there was tenderness in the left iliac region with no evidence of a mass. Hematological investigations were normal and urine pregnancy test was negative. Ultrasound Doppler revealed a 3.9 × 2.7 cm heterogenous mass in the left ovary with absent vascularity. A diagnostic laparoscopy revealed a twisted left fallopian tube at isthmic end (two twists) with ovary having a hemorrhagic cyst lying in POD (Figure 1(a)). Left tube was edematous, dilated, and congested with the fimbrial end appearing blue. Untwisting of the tube resulted in return of vascularity after 15 mins (Figure 1(b)). Postoperative period was uneventful. In the other case an 18-year-old patient presented with similar complaints since 1 week with aggravation of pain since two days. She had normal menstrual cycles with no past history of cyclical pain in abdomen, fever, or tuberculosis. There was mild tenderness in lower abdomen, and on per rectal examination a cystic tender mass of about 4 cm × 4 cm was felt in the midline. Ultrasonography (USG) revealed right adnexal cyst of 6.5 cm × 6.1 cm size with internal echoes. Hematological investigations and tumor markers were normal. Diagnostic laparoscopy showed a large terminally dilated twisted right fallopian tube (6 cm × 4 cm) which was dark blue and twisted two and a half times in a clockwise direction. Untwisting failed to improve color of tube and right salpingectomy was done (Figure 1(c)). Histopathological examination revealed a paratubal cyst lined by mesothelium. The fallopian tube and wall of the cyst showed marked congestion and hemorrhage.

## 3. Discussion

Isolated fallopian tube torsion is a rare event, occurring most commonly in the reproductive years and rarely in adolescents. While searching the literature for cases of isolated fallopian tube torsion in young girls, less than 40 reports were found on a PubMed search. In a recent review of 13 cases, the fallopian tube was lost in 11 [2]. The exact cause is not known but majority of the cases occur secondary to certain intrinsic and extrinsic tubal factors, which include abnormal length or spiral course of tube, hydrosalpinx, paraovarian cysts, pelvic adhesions, pelvic congestion, and pregnancy [3]. In our case an associated haemorrhagic ovarian cyst was probably the cause in the first and a paratubal cyst in the second case. It is generally unilateral with a predilection on right side [4]. It is suggested that this may be related to the presence of sigmoid colon on the left side or due to slow venous flow causing congestion and the greater willingness to explore right abdominal pain for appendicitis.

Sudden onset with sharp, colicky pelvic pain associated with nausea, vomiting, bowel, and bladder symptoms is the usual presentation [3]. The patient is hemodynamically stable with signs of peritonitis often absent at presentation. Imaging studies are nonspecific and ultrasound may show an elongated cystic mass near uterine cornu with ipsilateral ovary being separate from the mass. Finding of increased impedance or absent flow on Doppler in the tubal structure may indicate diagnosis [5]. Lack of pathognomonic clinical features and specific findings on radiological examination makes early preoperative diagnosis difficult, leading to delay in surgical intervention. MR imaging may be useful to suggest preoperative diagnosis and to depict hemorrhage related to irreversible disease. This again does not aid early preoperative diagnosis and intervention.

The diagnosis must be made early keeping a high clinical suspicion and laparoscopy or laparotomy undertaken as a matter of urgency. Timely intervention followed by detorsion of the fallopian tube could salvage it in the first case, and a delayed presentation in the second case resulted in loss of tube. Long term consequences of tubal detorsion need to be evaluated further in terms of future fertility and pregnancy outcome. However devitalized tissue should be removed as untwisting alone may cause thrombotic events [6]. 

## 4. Conclusion

Despite fallopian tube torsion being reported in literature, it continues to elude general gynecologists and precious tubes are still lost. Thus revisiting and reporting cases are important and should be kept as a differential diagnosis in all young women presenting with unilateral acute pelvic pain together with early intervention aimed at saving the tube. Laparoscopy remains the “gold standard” till superior imaging modalities are available.
